# Two-wavelength oximetry of tissue microcirculation based on sidestream dark-field imaging

**DOI:** 10.1117/1.JBO.24.3.031013

**Published:** 2018-10-30

**Authors:** Ryohei Hashimoto, Tomohiro Kurata, Masashi Sekine, Kazuya Nakano, Takashi Ohnishi, Hideaki Haneishi

**Affiliations:** a Chiba University, Graduate School of Science and Engineering, Chiba, Japan; b Takano Co., Ltd., Nagano, Japan; c Chiba University, Center for Frontier Medical Engineering, Chiba, Japan

**Keywords:** microcirculation, sidestream dark-field imaging, oximetry, hypoxia, oxygen saturation

## Abstract

Monitoring oxygen saturation (${\mathrm{SO}}_{2}$) in microcirculation is effective for understanding disease dynamics. We have developed an ${\mathrm{SO}}_{2}$ estimation method, sidestream dark-field (SDF) oximetry, based on SDF imaging. SDF imaging is a noninvasive and clinically applicable technique to observe microcirculation. We report the first *in vivo* experiment observing the changes in ${\mathrm{SO}}_{2}$ of microcirculation using SDF oximetry. First, heat from the light-emitting diodes used for the SDF imaging might affect hemodynamics in microcirculation, hence, we performed an experiment to evaluate the influence of that on the SDF oximetry. The result suggested that SDF oximetry had enough stability for long-term experiments. Then, to evaluate the sensitivity of SDF oximetry to alterations in the hemodynamics of the microcirculation, we observed the time-lapsed ${\mathrm{SO}}_{2}$ changes in the dermis microcirculation of rats under hypoxic stimulation. We confirmed that the ${\mathrm{SO}}_{2}$ estimated by SDF oximetry was in accordance with changes in the fraction of inspired oxygen (${\mathrm{FiO}}_{2}$). Thus, SDF oximetry is considered to be able to observe ${\mathrm{SO}}_{2}$ changes that occur in accordance with alteration of the microcirculation.

## 1 Introduction

Microcirculation is the blood flow through arterioles, capillaries, and venules, which exists throughout all tissues in a living body. Microcirculation plays an important role in supplying nutrients and oxygen to cells in the tissues. Thus, insufficient supplies of oxygen in microcirculation may lead to tissue hypoxia. Tissue hypoxia is considered to be one of the factors of serious diseases, such as cystic fibrosis and chronic bronchitis.^1^ Moreover, it is well known that varieties of experimental shock states (e.g., sepsis and haemorrhage) are related to hypoxia.^2^^–^^4^ In the early stages of these shock states, the impaired microcirculation will result in the rapid onset of tissue hypoxia. However, mechanisms of these diseases due to the hypoxia condition have not been clarified. Visualizing an individual microcirculation and observing the changes in the oxygen saturation (${\mathrm{SO}}_{2}$) during disease progression can be effective for understanding disease dynamics.

Many imaging techniques for ${\mathrm{SO}}_{2}$ in microcirculation have been developed. Snapshot multispectral imaging has been used for microcirculation oximetry mainly in the retina by using the difference in optical absorption properties of oxyhemoglobin (${\mathrm{HbO}}_{2}$) and deoxyhemoglobin (HbR).^5^ Photoacoustic imaging (PAI)^6^ and optical coherence tomography (OCT)^7^ also have been introduced to measure ${\mathrm{SO}}_{2}$ in microcirculation. However, these techniques require complex and expensive optical hardware. Therefore, simpler and lower cost optical setups would promote easier microcirculation assessment.

To visualize tissue microcirculation, orthogonal polarization spectral (OPS) imaging was developed as a real-time and noninvasive imaging technique of handheld vital microscopes (HVMs).^8^ This imaging technique was used to reveal alterations of the microcirculation in patients with sepsis.^9^ The second-generation HVM is based on sidestream dark-field (SDF) imaging.^10^^,^^11^ This imaging technique can acquire higher contrast microcirculation images than OPS imaging. From the SDF images of individual vessels, some physical quantities such as vessel length, vessel diameter, and velocity of red blood cells (RBCs) can be estimated.^12^ The first commercial SDF device (Microscan, Microvision Medical B.V., Amsterdam, The Netherlands) has been developed. To verify its effectiveness, evaluation experiments on patients with diabetes were performed.^11^^,^^13^ The results showed that the microcirculation density of patients with diabetes was significantly higher than that of healthy people. Compared with other imaging techniques of microcirculation such as PAI and OCT, SDF imaging is a simple and low cost technique. Hence, SDF imaging is easily applicable to clinical purposes and enables users to observe the microcirculation of human tissues.

Based on SDF imaging, we have developed a trial SDF probe and spectral image-based oximetry method, which we called SDF oximetry.^14^^,^^15^ Our SDF oximetry utilizes two-wavelength oximetry based on the Beer–Lambert law. In another study, we applied average extinction coefficients (AECs)^16^ of hemoglobin to SDF oximetry and improved the accuracy of our method. However, in our SDF oximetry, there are three problems that have to be overcome. First, we have already confirmed the effectiveness of SDF oximetry in tissue-like turbid phantom experiments; however, we have not carried out the *in vivo* experiments. Second, since we calculate the AECs from the SDF images of the turbid phantom experiments, they are not optimized for *in vivo* SDF images. Third, heat from the light-emitting diodes (LEDs) used for the SDF imaging might affect hemodynamics in microcirculation since the SDF probe is in direct contact with the tissue. We need to investigate the possible effect and that is the focus of this paper.

In this study, we conducted hypoxic stimulation experiments with rats by changing fraction of inspired oxygen (${\mathrm{FiO}}_{2}$). As a result, we confirmed that the estimated ${\mathrm{SO}}_{2}$ by SDF oximetry during the hypoxic stimulation experiment corresponded to the changes in ${\mathrm{FiO}}_{2}$. Prior to performing the *in vivo* experiment, we recalculated AECs from the literature values. Then, we examined the heat influence of the LEDs. Next, we conducted the experiment with rats where we observed the time-lapsed changes in ${\mathrm{SO}}_{2}$ under two conditions: normoxia and hypoxia. Then, we compared these ${\mathrm{SO}}_{2}$ values to evaluate our method, and the results suggested that SDF oximetry could response to alterations in microcirculation correctly.

## 2 Materials and Methods

### 2.1 SDF Imaging

**Fig. 1:**
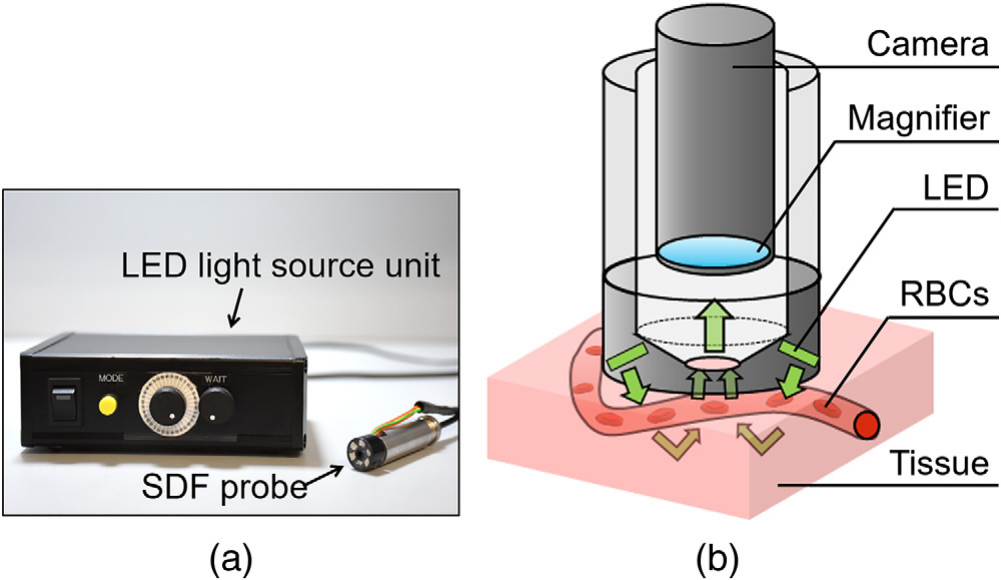
Overview of our trial SDF imaging system. (a) Photo of the SDF imaging setup. (b) Schematic illustration of an image acquisition.

**Fig. 2:**
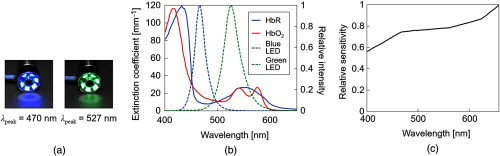
The two-color LEDs used in the SDF imaging system. (a) Photos of LEDs emitting light. The colors are blue for the 470-nm peak wavelength and green for the 527-nm one. (b) Spectral distribution of extinction coefficients of ${\mathrm{HbO}}_{2}$ and HbR (left axis) and spectral intensity distribution of the two-color LEDs with the relative intensity (right axis). The intensities of blue and green LEDs at the peak wavelength are 27 and $4.5\;\mathrm{cd}/m^{2}$, respectively. (c) Spectral sensitivity characteristics of the CMOS camera (relative value).

Figure 1 shows the overview of our trial SDF imaging system. Our system comprises a light source unit and SDF probe, as shown in Fig. 1(a). Figure 1(b) is a schematic illustration of an image acquisition. The SDF probe is in direct contact with the tissue and the pulsed illumination from the LEDs placed concentrically around the probe directly penetrates into the tissue. SDF imaging can only capture the light diffused in the tissues without surface reflections because the camera inside the SDF probe is optically isolated from the peripheral illumination. In SDF images, blood vessels are represented in black due to optical absorption by hemoglobin molecules of the RBCs. As an imaging device, a complementary metal oxide semiconductor (CMOS) camera (ID04MB-IP-U, iDule Corporation, Chiba, Japan) with a customized magnifier (focal length: 5.6 mm; F-number: F4.0) is used. Figure 2(a) shows the two-color LEDs (SMLVN6RGB1W, Rohm Co., Ltd., Kyoto, Japan) used in the system. The spectral intensities of the LEDs are shown in Fig. 2(b) together with extinction coefficients of ${\mathrm{HbO}}_{2}$ and HbR as a function of wavelength.^17^ The peak wavelengths of the two-color LEDs are 470 nm (blue) and 527 nm (green) and their full widths at half maximum are 27.3 and 37.2 nm, respectively. The wavelength band of the blue LED is sensitive to changes in hemoglobin oxygenation, whereas that of the green LED is insensitive. Therefore, we can acquire microcirculation images in different contrasts corresponding to hemoglobin oxygenation.

### 2.2 SDF Oximetry

**Fig. 3:**
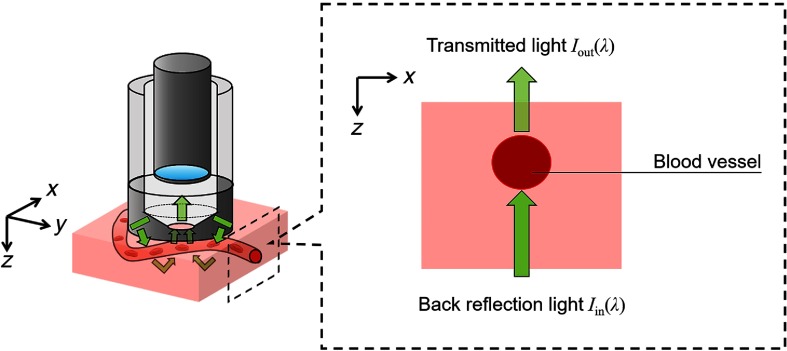
Assumption of $I_{\text{in}}(\lambda )$ and $I_{\text{out}}(\lambda )$ in the SDF imaging.

SDF oximetry utilizes two-wavelength oximetry based on the Beer–Lambert law.^14^ Figure 3 shows the assumption of $I_{\text{in}}(\lambda )$ and $I_{\text{out}}(\lambda )$ in the SDF imaging. We define the backreflection light entering into the blood vessel as the incident light $I_{\text{in}}(\lambda )$. When $I_{\text{in}}(\lambda )$ enters into the blood vessel with thickness d, the Beer–Lambert law gives the transmitted light $I_{\text{out}}(\lambda )$ through the medium as

$$I_{\text{out}}(\lambda )=I_{\mathrm{in}}(\lambda )\cdot \exp[-\varepsilon (\lambda )\cdot c\cdot d],$$ (1)

where ε(λ) represents the molar extinction coefficient of the hemoglobin and c represents the total hemoglobin concentration. Hemoglobin has several variants, namely methemoglobin (MetHb), carboxyhemoglobin (CoHb), and sulfhemoglobin (SulfHb). The absorption coefficients of these three hemoglobins have peaks in the range of visible light.^18^^,^^19^ However, concentrations of MetHb, CoHb, and SulfHb included in blood are normally lower than ${\mathrm{HbO}}_{2}$ and HbR, thus we can ignore them in our oximetry method. Moreover, absorption and scattering of leukocytes and platelets in blood occurs.^20^ However, these particles account for approximately only 4% of the whole blood volume, and hence they do not contribute more to absorption or scattering than RBCs do. As a result, for blood, ε(λ) is approximately defined as

$$\varepsilon (\lambda )={\mathrm{SO}}_{2}\cdot \varepsilon_{{\mathrm{HbO}}_{2}}(\lambda )+(1-{\mathrm{SO}}_{2})\varepsilon_{\mathrm{HbR}}(\lambda ),$$ (2)

where the ${\mathrm{SO}}_{2}$ range is 0 to 1. The optical density OD(λ) of blood is written as

$$\mathrm{OD}(\lambda )=-\log[\frac{I_{\mathrm{out}}(\lambda )}{I_{\mathrm{in}}(\lambda )}]=\varepsilon (\lambda )\cdot c\cdot d.$$ (3)

**Fig. 4:**
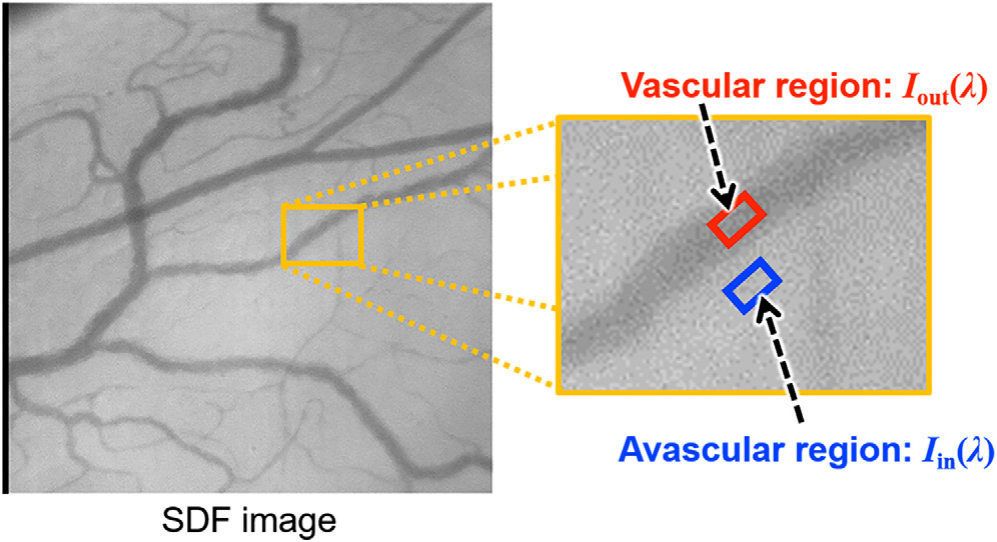
Definition of $I_{\text{in}}(\lambda )$ and $I_{\text{out}}(\lambda )$ in the SDF image.

Finally, solving two equations of Eq. (3) for $\lambda_{1}$ and $\lambda_{2}$, we obtain ${\mathrm{SO}}_{2}$ as follows:

$${\mathrm{SO}}_{2}=\frac{\mathrm{OD}(\lambda_{2})\cdot \varepsilon_{\mathrm{HbR}}(\lambda_{1})-\mathrm{OD}(\lambda_{1})\cdot \varepsilon_{\mathrm{HbR}}(\lambda_{2})}{\mathrm{OD}(\lambda_{1})\cdot \Delta \lambda_{2}-\mathrm{OD}(\lambda_{2})\cdot \Delta \lambda_{1}},$$ (4)

where $\Delta \lambda_{n}=\varepsilon_{\mathrm{HbO}2}(\lambda_{n})-\varepsilon_{\mathrm{HbR}}(\lambda_{n})$ for n=1,2. Figure 4 shows the definition of $I_{\text{in}}(\lambda )$ and $I_{\text{out}}(\lambda )$ in SDF images. In our definition, the intensity of vascular regions is treated as $I_{\text{out}}(\lambda )$ and that of avascular regions treated as $I_{\text{in}}(\lambda )$. Then, we can calculate ${\mathrm{SO}}_{2}$ by Eq. (4).

The ${\mathrm{SO}}_{2}$ value calculated by Eq. (4) cannot ignore the influence of the bandwidth of the LEDs shown in Fig. 2(b). We have to obtain the absorbance coefficients by wavelength averaging because the spectral distribution of the LEDs is not truly monochromatic. To take into account the bandwidth of the LEDs, we apply AECs ${\overset{¯}{\varepsilon }}_{\text{blue}}$ and ${\overset{¯}{\varepsilon }}_{\text{green}}$ instead of ε(λ).^14^ When the full width at 10th maximum is w, the intensity of light reaching the detector from vascular regions of ${\mathrm{HbO}}_{2}$ and HbR can be expressed as

$$I_{{\mathrm{out},\mathrm{HbO}}_{2},k}=\int_{\lambda_{k}-\frac{1}{2}w}^{\lambda_{k}+\frac{1}{2}w}S(\lambda )\cdot L_{k}(\lambda )\cdot \exp[-\varepsilon_{{\mathrm{HbO}}_{2}}(\lambda )\cdot c\cdot d]d\lambda ,$$ (5)

$$I_{\mathrm{out},\mathrm{HbR},k}=\int_{\lambda_{k}-\frac{1}{2}w}^{\lambda_{k}+\frac{1}{2}w}S(\lambda )\cdot L_{k}(\lambda )\cdot \exp[-\varepsilon_{\mathrm{HbR}}(\lambda )\cdot c\cdot d]d\lambda ,$$ (6)

where S(λ) represents the spectral sensitivity of the camera and $L_{k}(\lambda )$ represents the spectral distribution of the k’th color of the LEDs. The intensity of light reaching the detector from avascular regions is expressed as

$$I_{\mathrm{in},k}=\int_{\lambda_{k}-\frac{1}{2}w}^{\lambda_{k}+\frac{1}{2}w}S(\lambda )\cdot L_{k}(\lambda )d\lambda .$$ (7)

Hence, the AECs of ${\mathrm{HbO}}_{2}$ and HbR can be defined as

$${\overset{¯}{\varepsilon }}_{{\mathrm{HbO}}_{2},k}=-\frac{1}{cd}\log(\frac{I_{{\mathrm{out},\mathrm{HbO}}_{2},k}}{I_{\mathrm{in},k}}),$$ (8)

$${\overset{¯}{\varepsilon }}_{\mathrm{HbR},k}=-\frac{1}{cd}\log(\frac{I_{\mathrm{out},\mathrm{Hb},k}}{I_{\mathrm{in},k}}).$$ (9)

We calculated these AECs from the spectral distribution of the extinction coefficients of ${\mathrm{HbO}}_{2}$ and HbR,^14^ the spectral intensity distribution of the two-color LEDs, and the spectral sensitivity characteristics of the CMOS camera, shown in Figs. 2(b) and 2(c). The calculated results were $cd{\overset{¯}{\varepsilon }}_{\mathrm{HbO}2,\mathrm{blue}}=13.5\;{\mathrm{mm}}^{-1}$, $cd{\overset{¯}{\varepsilon }}_{\mathrm{HbR},\mathrm{blue}}=8.47\;{\mathrm{mm}}^{-1}$, $cd{\overset{¯}{\varepsilon }}_{\mathrm{HbO}2,\text{green}}=11.2\;{\mathrm{mm}}^{-1}$, and $cd{\overset{¯}{\varepsilon }}_{\mathrm{HbR},\text{green}}=11.2\;{\mathrm{mm}}^{-1}$.

### 2.3 In Vivo Imaging Preparation

**Fig. 5:**
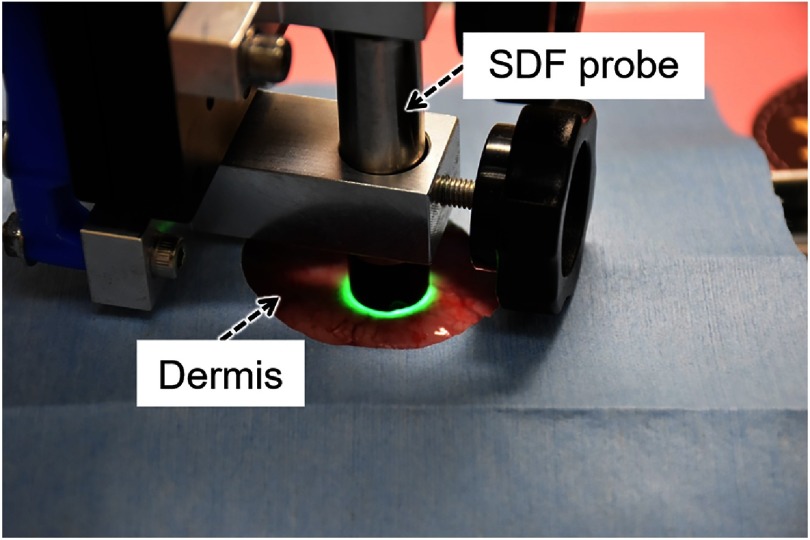
*In vivo* experiment setup for SDF imaging.

Figure 5 is a photo of the experiment setup. In our experiment, we observed dermis microcirculation of male Slc:Wistar rats (260 to 280 g, 12-weeks old, n=3). One was used for an experiment of stability evaluation, and the other two were for a hypoxic stimulation experiment. These rats were anesthetized under 2.0% isoflurane (Escain, Pfizer, Tokyo, Japan). A heat mat (Kainuma Industrial Co., Ltd., Aichi, Japan) was laid under the rat to help maintain its body temperature. To obtain clear images, the epidermis was removed before the observation. To monitor the percutaneous arterial oxygen saturation (${\mathrm{SpO}}_{2}$), a rat pulse oximeter (MouseOx Plus, STARR Life, Pennsylvania) was used.

As the first experiment, to ensure the time-lapsed changes in ${\mathrm{SO}}_{2}$ of hypoxic stimulation are not influenced by the heat from LEDs, a rat (rat 1) was observed during an 11-min period under normoxia. The rat’s mouth and nose were covered with a mask that was connected to an inhalation tube. The SDF images were acquired under blue and green illuminations alternately every 100 ms. Next, to assess the sensitivity of the SDF oximetry in accordance with physiological changes, 2 rats (rat 2 and 3) were exposed to hypoxic stimulation by changing ${\mathrm{FiO}}_{2}$. Similar procedures have been employed in previous studies.^21^^,^^22^ They were kept to inhale the air mixed with isoflurane for the first 3 min. The oxygen concentration was then reduced to 10% oxygen by increasing nitrogen concentration together with isoflurane for 3 min to get the hypoxic stimulus state, which was followed by a switch back to the normal state for 5 min. The SDF images were acquired in the same manner as done in the first experiment. All experiments in this study were carried out in conformity with the Institutional Animal Care and Use policy of Chiba University.

## 3 Experiments and Discussion

### 3.1 Time-Lapsed Stability Evaluation of SDF Oximetry

The hypoxic stimulation experiment, described in Sec. 3.2, lasted 11 min. Due to the long measurement time, there are two possible influences on the estimated ${\mathrm{SO}}_{2}$. The first is the heat influence caused by the LEDs. In SDF imaging, the SDF probe in which the LEDs are arranged is in direct contact with the tissue. Hence, the self-heating of LEDs caused by continuous light emission may affect microcirculation hemodynamics. The second is the influence of isoflurane. For example, Helmchen et al.^23^ observed anesthetic-induced changes in microcirculation hemodynamics. Therefore, we have to evaluate the stability of SDF oximetry during an 11-min period to ensure that time-lapsed changes in ${\mathrm{SO}}_{2}$ of hypoxic stimulation are not influenced by these two factors.

**Fig. 6:**
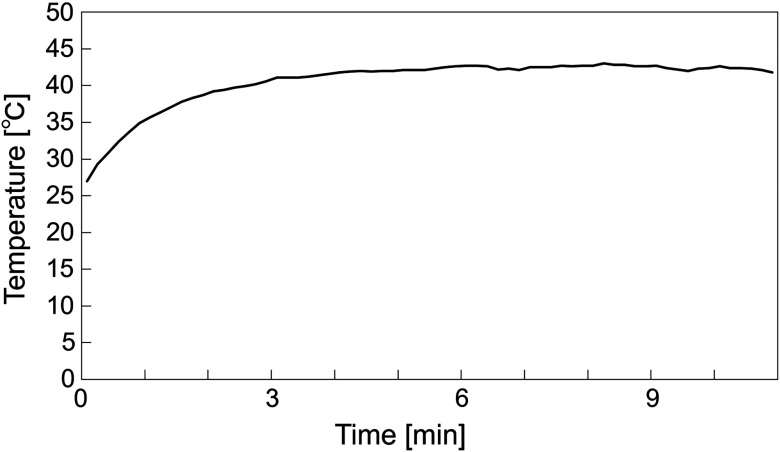
Time-lapsed changes in the temperature of SDF probe surface.

**Fig. 7:**
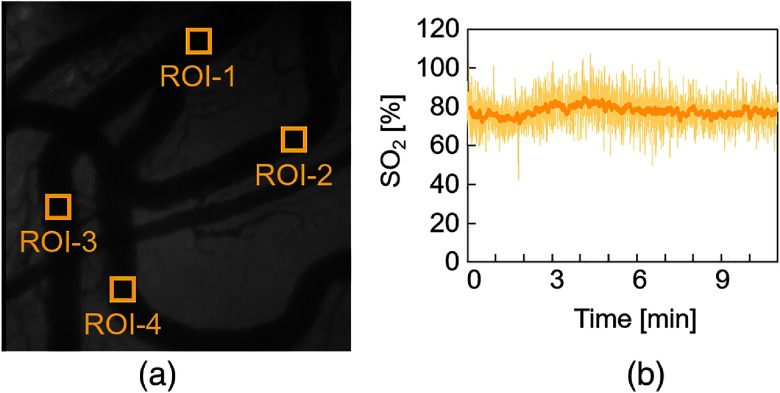
Stability evaluation of our SDF oximetry. (Rat 1) (a) SDF image obtained under blue LED illumination at 0 min. The labels ROI-1 to ROI-4 represent the regions of interest for ${\mathrm{SO}}_{2}$ estimation. (b) Time-lapsed changes in ${\mathrm{SO}}_{2}$ during 11 min for ROI-1. The orange solid line indicates the moving average for 6 s.

Prior to performing the *in vivo* experiment, we measured the time-lapsed changes in the temperature of the surface of SDF probe. We used a thermometer (AM-8051E, Anritsu Meter Co., Ltd., Tokyo, Japan) with a flexible internal temperature sensor (SF-E-200-ANP, Anritsu Meter Co., Ltd., Tokyo, Japan) for temperature measurement. As shown in Fig. 6, we found that the temperature of the SDF probe surface gradually increased and reached ∼42°C. Then, we performed the *in vivo* experiment to evaluate the stability of SDF oximetry during an 11-min period. Figure 7(a) shows the SDF image obtained under the blue LED illumination at 0 min. We analyzed the time-lapsed changes in ${\mathrm{SO}}_{2}$ calculated by Eq. (4) for each region of interest (ROI) and calculated mean and standard deviation as shown in Fig. 7(b) and Table 1. The figure and table showed that there were no significant changes in ${\mathrm{SO}}_{2}$ during 11 min. The results indicated the time-lapsed stability of our SDF oximetry. It means that in an 11 min-long experiment, there is no influence of self-heating of the LEDs and isoflurane. In Fig. 7(b), large variations in the estimated value of ${\mathrm{SO}}_{2}$ are observed. It is because that the two SDF images were acquired under blue and green illuminations alternately every 100 ms. Since these images have a time-lag, each of their corresponding pixels used for ${\mathrm{SO}}_{2}$ estimation does not contain the exact same density of RBCs. If the density of RBCs within the pixel differs between two acquired SDF images, it results in an over- or underestimation of ${\mathrm{SO}}_{2}$. However, adopting a moving average, we can reduce the estimation error.

**Table 1:** Mean ± standard deviation (%) of estimated ${\mathrm{SO}}_{2}$ (rat 1).

Region of interest			
ROI-1	ROI-2	ROI-3	ROI-4
77.7±2.4	90.0±4.8	87.0±3.7	82.6±5.6

### 3.2 Observation of Time-Lapsed Changes in ${\mathrm{SO}}_{2}$ During Hypoxic Stimulation

**Fig. 8:**
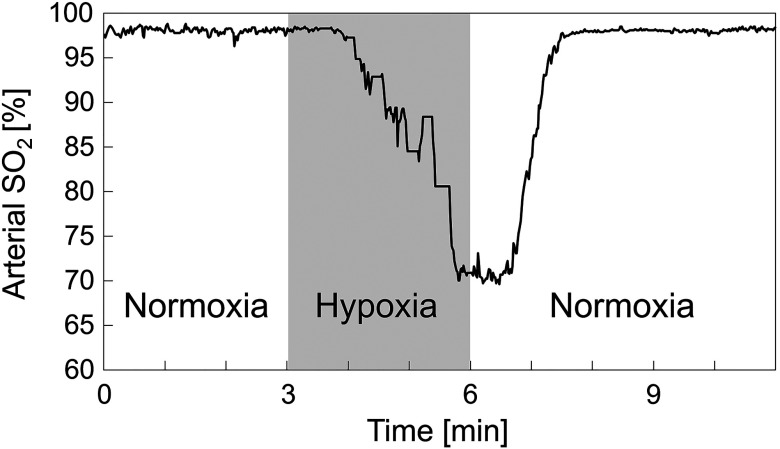
Response of the ${\mathrm{SpO}}_{2}$ measured by the pulse oximeter during the experiment (rat 2).

**Fig. 9:**
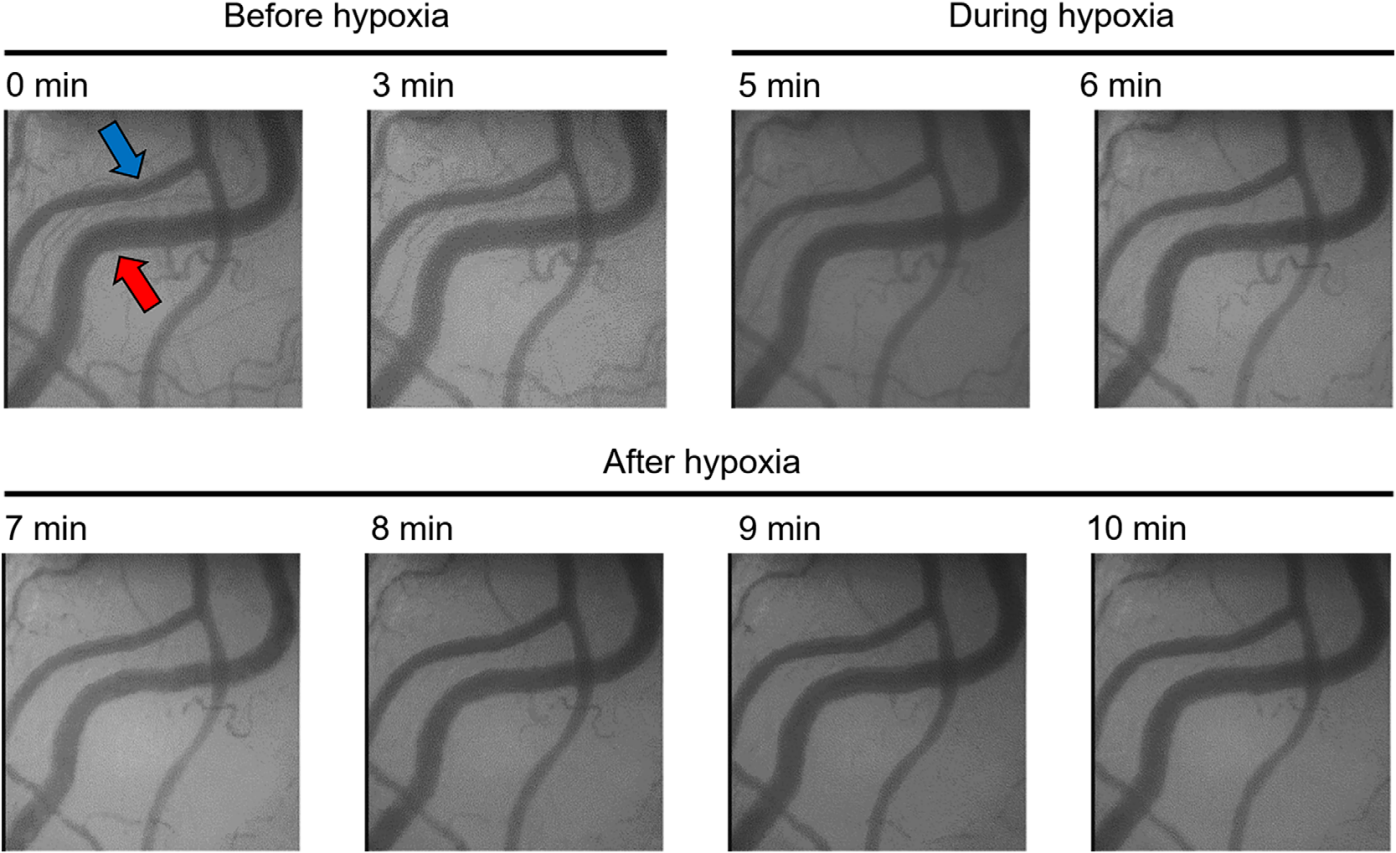
Time-lapsed SDF images during hypoxic stimulation experiment (rat 2).

The hypoxic stimulation experiment in this section was repeated using two rats. We describe results for rat 2 first. As shown in Fig. 8, the response of the ${\mathrm{SpO}}_{2}$ measured by the pulse oximeter during the hypoxic stimulation experiment corresponded to the change in ${\mathrm{FiO}}_{2}$. The decrease and increase in ${\mathrm{SpO}}_{2}$ started ∼1  min after the change in ${\mathrm{FiO}}_{2}$. Figure 9 shows the time-lapsed SDF images. We could observe a slight vasoconstriction and a decrease in blood flow in accordance with the hypoxic stimulation. The vasoconstriction is caused by temporary elevation of blood pressure associated with hypoxia.^24^ After hypoxia, these two characteristics returned to their original state. In the ${\mathrm{SO}}_{2}$ analysis, we focused on two blood vessels. One had a bifurcation in the field of view (blue arrow) and the other had no bifurcation (red arrow). In particular, we predicted that the blood vessel indicated by the blue arrow was the venule because the blood flow was merged at the vessel branch.

**Fig. 10:**
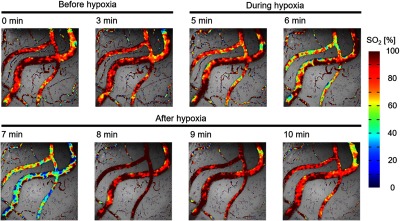
Alterations of ${\mathrm{SO}}_{2}$ maps during hypoxic stimulation (rat 2).

**Fig. 11:**
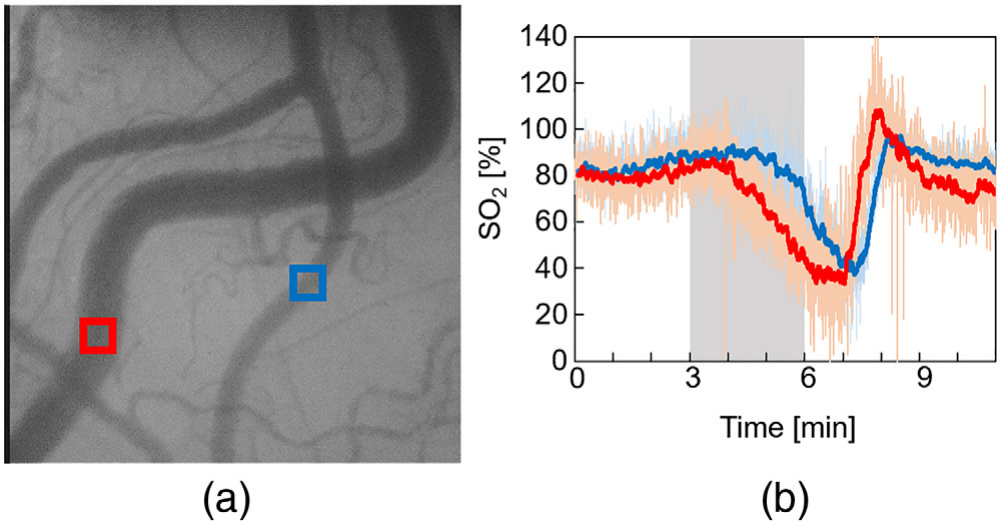
Changes in estimated ${\mathrm{SO}}_{2}$ during hypoxic stimulation (rat 2). (a) Obtained SDF image. (b) Time-lapsed changes in ${\mathrm{SO}}_{2}$ during hypoxic stimulation. The red and blue solid lines indicate the moving average for 6 s.

${\mathrm{SO}}_{2}$ of individual vessels were calculated for each pixel from two-band SDF images. Figure 10 shows the changes in ${\mathrm{SO}}_{2}$ maps during hypoxic stimulation. The results clearly showed that the ${\mathrm{SO}}_{2}$ in microcirculation decreased during hypoxic stimulation and increased after hypoxia. Figure 11(b) shows the changes in estimated ${\mathrm{SO}}_{2}$ during hypoxic stimulation corresponding to the rectangular area shown in Fig. 11(a). The size of the area was set to 20×20  pixels. The gray-shaded area meant the period of hypoxic stimulation. These results mostly corresponded to the change in the ${\mathrm{SpO}}_{2}$, measured by the pulse oximeter shown in Fig. 8. After returning to the normoxia stimulation, the estimated ${\mathrm{SO}}_{2}$ increased nearly 100% almost instantaneously and then decreased gradually. This result might reflect the function of microcirculation to regulate the tissue oxygenation.

Further, we focused on the difference in response to hypoxia between two blood vessels, which are marked by the red and blue arrows in Fig. 11. As shown in Fig. 11(b), the ${\mathrm{SO}}_{2}$ of the blue rectangular area began to change before there were observable changes in the red rectangular area. These differences between the two blood vessels may be due to the difference between arterioles and venules as mentioned in a previous study.^5^ In SDF imaging, arterioles and venules are distinguished by the blood flow direction at the vessel branch. However, more accurate identification requires a larger image size than that of the present SDF images ($0.8\times 0.8\;{\mathrm{mm}}^{2}$).

**Fig. 12:**
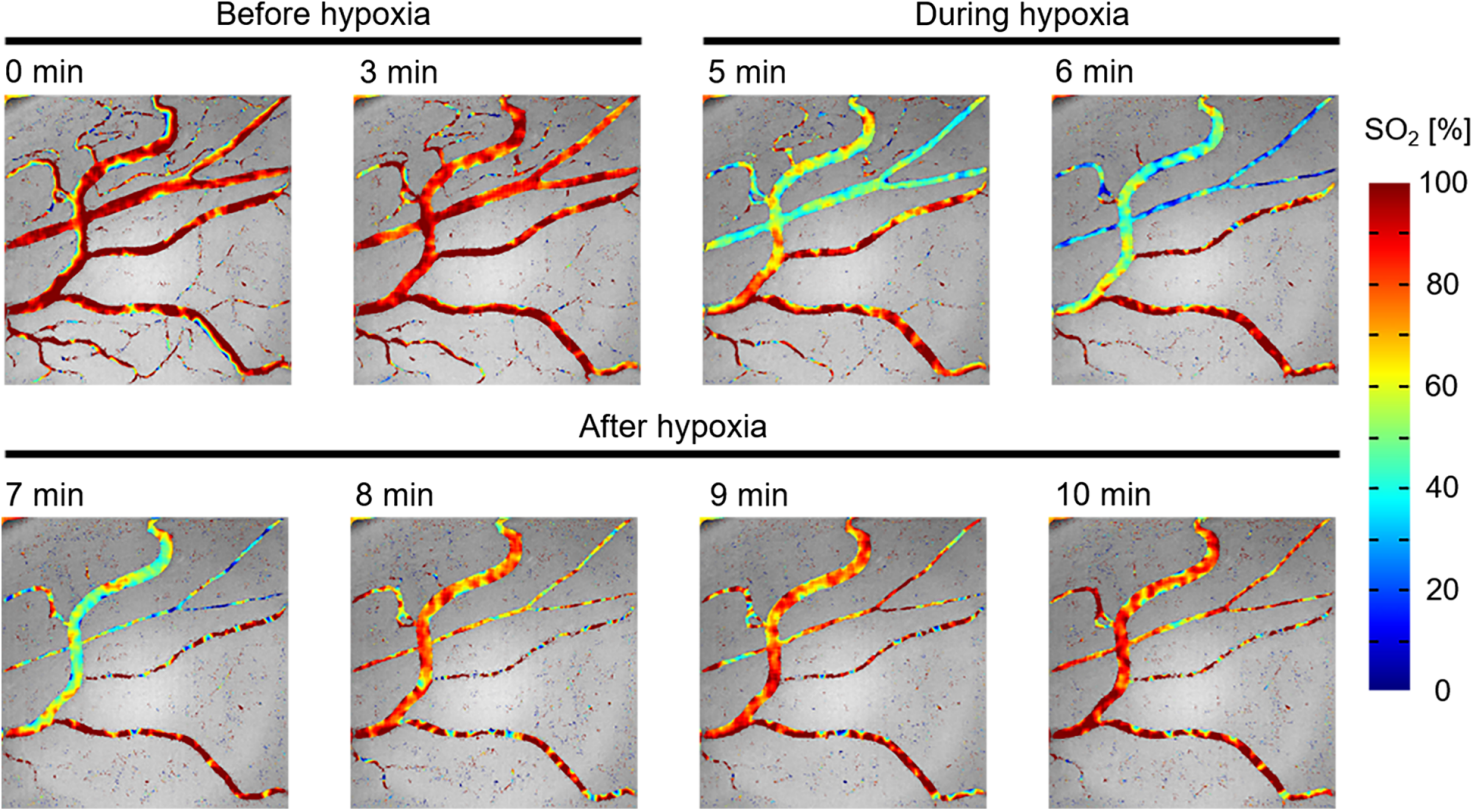
Alterations of ${\mathrm{SO}}_{2}$ maps during hypoxic stimulation (rat 3).

**Fig. 13:**
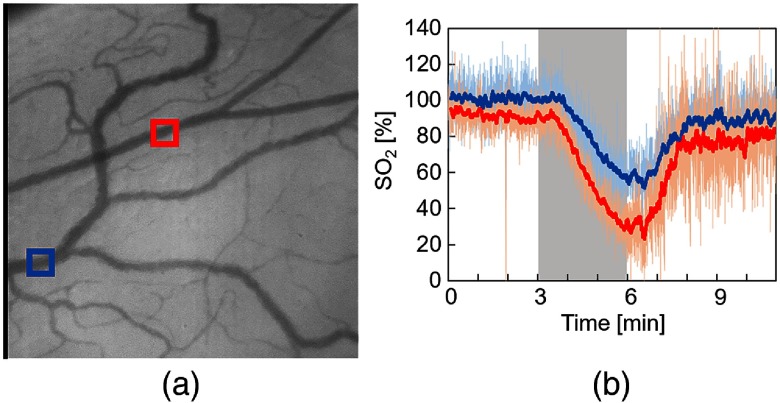
Changes in estimated ${\mathrm{SO}}_{2}$ during hypoxic stimulation (rat 3). (a) Obtained SDF image. (b) Time-lapsed changes in ${\mathrm{SO}}_{2}$ during hypoxic stimulation. The red and blue solid lines indicate the moving average for 6 s.

**Fig. 14:**
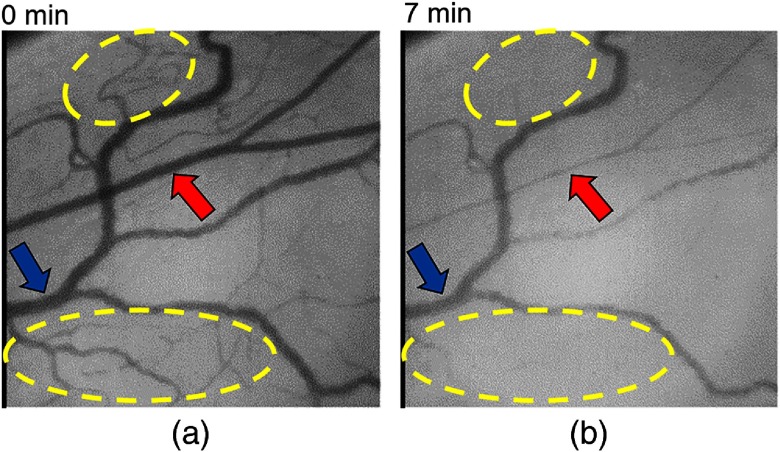
Comparison of blood vessel structure between before and after hypoxia (rat 3).

In order to ensure the reproducibility of the hypoxic stimulation experiment, we repeated it with the other rat (rat 3). The experimental conditions were the same as the first time. Figure 12 shows that the created ${\mathrm{SO}}_{2}$ maps had a tendency similar to that of Fig. 10. In addition, the difference in the change of timing between different vessels was also observed as shown in Fig. 13. In the repeated experiment, the response speed for the estimated ${\mathrm{SO}}_{2}$ to hypoxia was faster than that of the first. We considered that to be due to individual differences between the rats or to differences of the blood vessel diameters. Figure 14 shows SDF images obtained at 0 and 7 min. There were two characteristics. First, the blood vessel indicated by the red arrow decreased in diameter compared with that indicated by the blue arrow. To compensate for the insufficient oxygen supply, the diameter of the arterioles presumably varied for the increasing blood pressure. Second, blood vessel density in the areas indicated by the yellow circles was reduced by hypoxia. We considered that the decrease in blood flow caused by hypoxia resulted in the lack of perfusion to capillaries and the reduction of blood vessel density.

Our results showed that SDF oximetry could estimate ${\mathrm{SO}}_{2}$ of microcirculation and respond to an alteration in microcirculation in hypoxic stimulation experiments with rats. This indicates that SDF oximetry can be applied for understanding microcirculatory dysfunction in oxygen delivery induced by several diseases, as mentioned in the introduction. However, the accuracy of absolute ${\mathrm{SO}}_{2}$ value estimated by SDF oximetry is uncertain. Therefore, we have to compare the ${\mathrm{SO}}_{2}$ value estimated by SDF oximetry with the one obtained by using high accuracy oximeter (e.g., blood gas analyzer).

## 4 Conclusion

In this paper, we have applied our SDF oximetry to *in vivo* experiments and observed the time-lapsed SO_2_ changes in dermis microcirculation of rats under hypoxic stimulation. Before performing the *in vivo* experiment, we calculated the AECs from the literature values because the AECs used in our previous study were optimized only for turbid phantom experiments. Next, to confirm the time-lapsed stability of SDF oximetry, we obtained SDF images under the normoxia during an 11-min period. The results suggested that hemodynamics in microcirculation were not affected by the heat from the LEDs. Then, we conducted the *in vivo* experiment with rats to evaluate the sensitivity of SDF oximetry under hypoxic stimulation. This is the first *in vivo* experiment that observed the changes in ${\mathrm{SO}}_{2}$ of microcirculation based on SDF imaging.

We confirmed that the estimated ${\mathrm{SO}}_{2}$ by SDF oximetry was in accordance with the change in ${\mathrm{FiO}}_{2}$. Therefore, SDF oximetry is considered to be a method that enables us to observe ${\mathrm{SO}}_{2}$ changes occurring in accordance with alteration of microcirculation. Moreover, we focused on not only the overall ${\mathrm{SO}}_{2}$ changes in microcirculation but also the difference between individual blood vessels. The speeds of these responses to hypoxic stimulation were different. These differences were thought to be due to the difference between arterioles and venules as reported previously,^5^ or to differences of blood vessel diameter.

To raise the reliability of ${\mathrm{SO}}_{2}$ estimations, future work must take into account the error factors of SDF oximetry such as the influence of scattering by tissues, as mentioned in our previous study.^15^ As one solution to this problem, Monte Carlo simulation of photon propagation is considered. Analyzing the photon propagation in the tissue would enable researchers to understand the behavior of photons and lessen the extent of the error factors. After improving the accuracy of ${\mathrm{SO}}_{2}$ estimation, as future work, we will conduct *in vivo* experiments with a sepsis rat model by following the same procedure as described in our previous work.^25^ Then, we will investigate the relationship between oxygen delivery and mechanism of sepsis shock.
